# Type 2 diabetes burden among migrants in Europe: unravelling the causal pathways

**DOI:** 10.1007/s00125-021-05586-1

**Published:** 2021-10-16

**Authors:** Charles Agyemang, Eva L. van der Linden, Louise Bennet

**Affiliations:** 1 Department of Public & Occupational Health, Amsterdam Public Health Research Institute, Amsterdam University Medical Centers, University of Amsterdam, Amsterdam, the Netherlands; 2 Department of Vascular Medicine, Amsterdam Cardiovascular Sciences, Amsterdam University Medical Centers, University of Amsterdam, Amsterdam, the Netherlands; 3 Department of Clinical Sciences in Malmö, Lund University, Malmö, Sweden; 4 Clinical Research and Trial Centre, Lund University Hospital, Lund, Sweden

**Keywords:** Ethnic minority groups, Europe, Migrants, Review, Type 2 diabetes

## Abstract

European populations are ethnically and culturally diverse due to international migration. Evidence indicates large ethnic inequalities in the prevalence of type 2 diabetes. This review discusses the burden of type 2 diabetes and its related complications, and the potential explanatory mechanisms among migrants in Europe. The current available data suggest that the rate of type 2 diabetes is higher in all migrant groups and that they develop this disease at an earlier age than the host European populations. The level of diabetes awareness among migrant populations is high, but glycaemic control remains suboptimal compared with Europeans. The culturally adapted lifestyle modification intervention trials to prevent type 2 diabetes mainly focus on South Asian adults in Europe. Diabetes-related microvascular and macrovascular complications remain a major burden among migrant populations in Europe. Earlier studies found higher mortality rates among migrants, but recent studies seem to suggest a shifting trend in favour of first-generation migrants. However, the extent of the burden of type 2 diabetes varies across migrant groups and European countries. Despite the higher burden of type 2 diabetes among migrants, the key underlying factors are not well understood mainly due to limited investment in basic science research and development of prospective cohort studies. We hypothesise that the underlying risk factors for the high burden of type 2 diabetes and its related complications in migrants are multifaceted and include pre-migration factors, post-migration factors and genetic predispositions. Given the multi-ethnic nature of the current European population, there is a clear need for investment in research among migrant populations to gain insight into factors driving the high burden of type 2 diabetes and related complications to facilitate prevention and treatment efforts in Europe.

**Graphical abstract:**

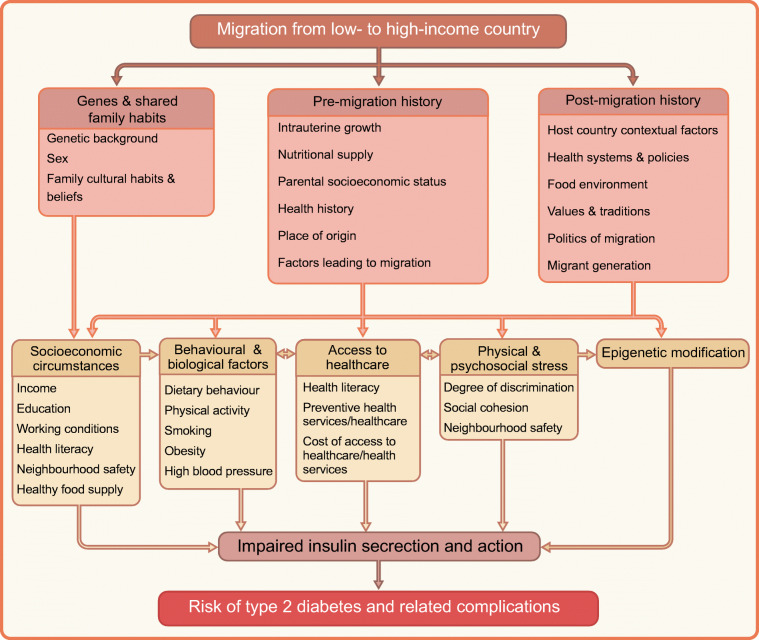

**Supplementary Information:**

The online version contains a slideset of the figures for download, which is available to authorised users, available at 10.1007/s00125-021-05586-1.

## Introduction

Type 2 diabetes is one of the leading causes of cardiovascular morbidity and mortality and contributes to an increasing disease burden in all European countries [1]. Since the 1990s, the prevalence of type 2 diabetes has increased steadily in Europe, and estimates suggest that the age-adjusted prevalence rate is expected to rise from 6.3% in 2019 to 7.8% in 2045 [2]. Evidence indicates large inequalities in the rates of diabetes and related complications between migrant and ethnic minority groups (henceforth, ‘migrants’) and the host European-origin populations (henceforth, ‘Europeans’) [3]. Europe is ethnically diverse, especially in the urban centres, due to international migration. In the last few decades, political instability, particularly in low-resource regions of the world, has forced millions of people to seek a more stable future outside their home countries. Europe remains one of the main recipients of migrants from low-resource regions of the world, and migration is expected to increase in the coming decades [4]. To prevent early disease onset and diabetes-related complications in migrants in Europe, special attention is needed to identify high-risk populations and the underlying determinants to guide prevention and treatment efforts. Drawing on evidence from the existing literature, this review discusses the burden of type 2 diabetes and its related complications and the potential explanatory mechanisms in migrants in Europe. The review also discusses evidence on prevention and treatment of diabetes in migrants and presents recommendations for addressing future challenges in Europe with special emphasis on migrants from low-resource countries. 
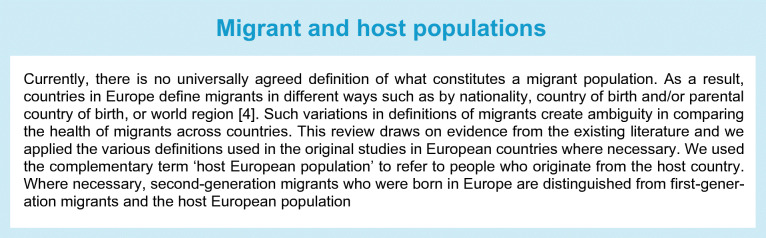


## Type 2 diabetes burden in migrants

**Fig. 1:**
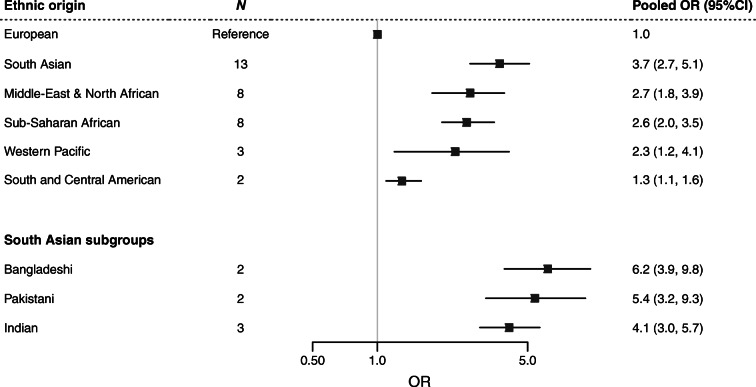
Type 2 diabetes in ethnic minorities in Europe. Pooled ORs (95% CIs) of type 2 diabetes in ethnic minorities in Europe compared with the host European population are shown. *N* indicates the number of studies covering this population group. Data source: Meeks et al. [3]. This figure is available as part of a downloadable slideset

**Fig. 2:**
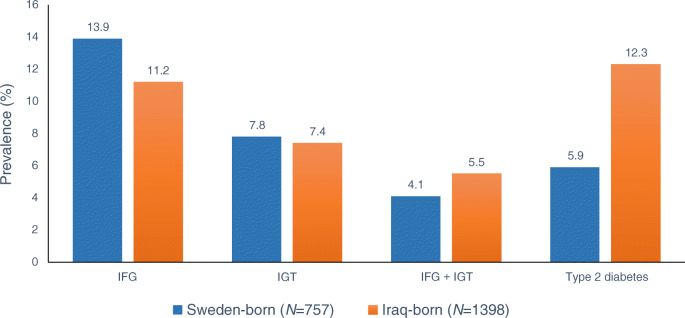
Prevalence of prediabetes (IFG, IGT, or both) and type 2 diabetes in the MEDIM cohort, showing data for Middle Eastern migrants and the host European population of Swedes. Data source: Bennet et al. [7]. This figure is available as part of a downloadable slideset

The prevalence of type 2 diabetes differs importantly between ethnic groups in Europe. A meta-analysis of its prevalence among migrant groups in Europe, of papers published between 1994 and 2014, shows that generally, these groups had higher rates of type 2 diabetes compared with Europeans [3]. Migrants from South Asia had the highest rate, with the pooled OR being nearly fourfold higher, followed by those from the Middle East and North Africa, Sub-Saharan Africa and South and Central America, compared with Europeans (Fig. 1). Type 2 diabetes prevalence also differed importantly between people from a similar region of origin. For example, among the South Asian subgroups, the pooled OR of type 2 diabetes was sixfold higher in Bangladeshis, fivefold higher in Pakistanis and fourfold higher in Indians compared with Europeans [3]. The meta-analysis also shows a clear sex difference, with the magnitude of the ethnic differences in type 2 diabetes being greater in migrant women than in men, relative to Europeans [3]. Furthermore, several studies have found a higher prevalence of prediabetes or impaired fasting glucose (IFG) in several migrant groups, including migrant children, compared with Europeans [5, 6]. Middle Eastern migrants in Sweden have been shown to have higher prevalence rates of insulin resistance [7] and combined IFG&IGT than Europeans (Fig. 2), predisposing them to a high risk of developing diabetes.

Data on intergenerational differences in type 2 diabetes prevalence among migrants are scarce but seem to suggest that the burden of diabetes cuts across different generations of migrants. A recent study among three generations of Moluccans resident in the Netherlands found higher odds of type 2 diabetes across all the three generations compared with European Dutch [8].

Evidence indicates that migrants develop type 2 diabetes 10–20 years earlier [9–11] and at a lower level of BMI [12, 13] than Europeans. The United Kingdom (UK) Biobank cohort data, for example, show that, for the equivalent prevalence of diabetes at 30 kg/m^2^ in European descent participants, BMI equated to 22.0 kg/m^2^ in South Asians, 26.0 kg/m^2^ in African descent populations and 24.0 kg/m^2^ and 26.0 kg/m^2^, respectively, in Chinese women and Chinese men [12]. Diabetes prevalence was also found to be higher in migrant children (<16 years) than in European children in the UK [14]. Diabetes precursors, including HbA_1c_, insulin, triacylglycerol and C-reactive protein levels, have also been shown to be higher in South Asian and African-Caribbean children than in European children, even after adjusting for differences in adiposity in the UK [15, 16].

The prevalence of type 2 diabetes among migrants in Europe is also far higher than among the populations in their countries of origin. For instance, findings from the Research on Obesity and Diabetes among African Migrants (RODAM) study show that age- and education-adjusted prevalence of type 2 diabetes in men ranges from threefold higher in Ghanaian migrants in London to nearly fivefold higher in Ghanaian migrants in Berlin compared with their rural Ghanaian peers [17]. In addition, Bhatnager et al. found that Punjabi people living in West London had higher fasting blood glucose than their siblings living in the Punjab [18]. Furthermore, Mbanya et al. found among populations of African descent an increased gradient of type 2 diabetes prevalence, from rural Cameroon (0.8%) to urban Cameroon (2.0%) through Jamaica (8.5%) to Manchester, UK (14.6%) [19].

## Diabetes-related complications in migrants

### Complications

The prevalence of diabetes-related complications also differs between migrant groups and Europeans [10, 20]. Evidence indicates a high risk of microvascular and macrovascular complications in migrants with type 2 diabetes than their European counterparts [21–23] but with great variations across migrant populations. In the recent Healthy Life in an Urban Setting (HELIUS) study, South Asian Surinamese, Moroccan and Turkish migrants had age- and sex-adjusted higher odds of nephropathy than European Dutch. The rate of coronary heart disease (CHD) was also higher in all migrant groups, with ORs ranging from nearly threefold higher in Ghanaian migrants to nearly sevenfold higher in Turkish migrants compared with European Dutch in the fully adjusted model [21]. In an 11-year follow-up of the Southall Diabetes Study, South Asians with type 2 diabetes were almost fourfold more likely than Europeans to report a history of myocardial infarction, but there was no significant difference in the risk of hypertension, stroke or amputation [10]. Furthermore, a 20-year follow-up of the UK-based Southall And Brent REvisited (SABRE) study showed that the risk of stroke was almost twice as high in South Asians and over twofold higher in African-Caribbean individuals with diabetes compared with their European peers [24]. A higher CHD rate was also observed in Iraqi migrants compared with Europeans in the All New Diabetics In Scania (ANDIS) study, a 10-year follow-up of individuals with new-onset type 2 diabetes in Sweden [25]. In contrast, the same study found a considerably lower incidence of chronic kidney disease in Iraqi migrants compared with European Swedes.

### Mortality risk

**Fig. 3:**
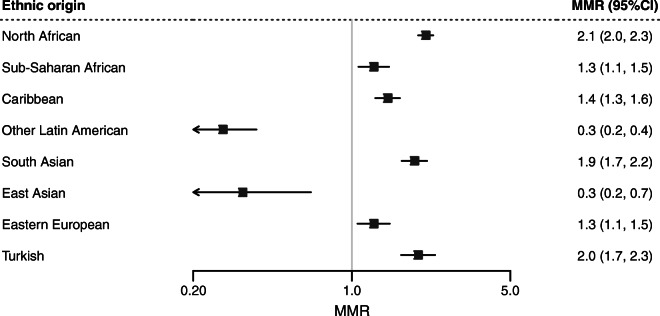
Mortality rate ratios (MRRs) and 95% CIs in diabetes-related mortality among migrant groups compared with Europeans in six European countries (Denmark, England and Wales, France, the Netherlands, Scotland, and Spain). MRRs were adjusted for sex, age and country of destination. Data source: Ikram et al. [26]. This figure is available as part of a downloadable slideset

The data on ethnic differences in diabetes-related mortality remain complex. In one study that examined differences in causes of death across different migrant groups and Europeans living in six European countries, the diabetes mortality rate ratio was higher for most migrant populations born in North Africa, Sub-Saharan Africa, the Caribbean, South Asia and Turkey but was lower for those born in East Asia and Latin America (Fig. 3) [26]. These findings corroborate those of earlier studies showing the relatively higher risk of death among migrants with type 2 diabetes compared with Europeans [10, 27]. By contrast, more recent studies show lower mortality risks in migrants with diabetes compared with Europeans [28]. In a nationwide 10-year longitudinal study of type 2 diabetes in Sweden, the adjusted risk of all-cause mortality and cause-specific mortality (due to CVD or cancer) was lower in first-generation migrants born in the Middle East, Asia, Africa and Latin America compared with European Swedes [11]. However, second-generation migrants with type 2 diabetes, especially those with both parents born abroad, had a 28% higher risk of all-cause mortality than the European Swedes [11]. In another recent cohort study using Clinical Practice Research Datalink data from 383 general practices in England, the adjusted risks for mortality from CVD, cancer and respiratory diseases were lower in people of South Asian and African descent with diabetes compared with European with diabetes [28]. A lower risk of death has also been observed among South Asian and Chinese people with diabetes in Canada [29]. The emerging mortality advantage among migrants with type 2 diabetes is surprising and difficult to explain especially given their relatively high risk of microvascular and macrovascular complications [21–23]. It is probable that this mortality advantage may be explained, at least in part, by biological or lifestyle factors that contribute to low mortality, such as low epigenetic age acceleration [30] or beneficial effect of lifestyle intervention and medication at a young age in migrants as they appear to develop type 2 diabetes earlier than Europeans [28].

## Potential explanatory mechanisms for the type 2 diabetes burden in migrants

**Fig. 4:**
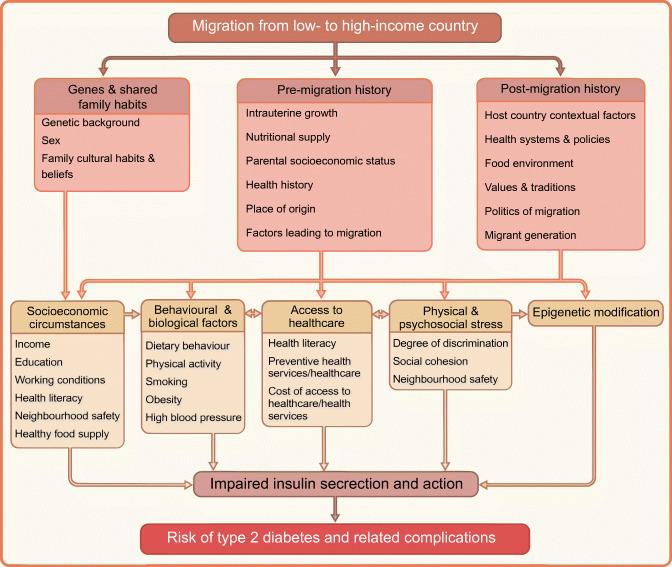
Pathways leading to the high risk of type 2 diabetes in migrants. This figure is available as part of a downloadable slideset

Despite the high prevalence of diabetes and its related complications among migrants in Europe, the key underlying drivers are still not well understood. This has been mainly attributed to the inadequate investment in basic scientific research into pathophysiology and longitudinal epidemiological studies to test the relevant hypotheses in these populations. Nevertheless, it is well acknowledged that the potential factors underlying the high risk of type 2 diabetes and its related complications in migrants are multifaceted, including post-migration factors [31], pre-migration factors and genetic predispositions [32]. The conceptual model of hypothesised causal pathways leading to the high risk of type 2 diabetes in migrants is shown in Fig. 4. It is hypothesised that pre-migration factors (e.g. intrauterine growth, parental socioeconomic status [SES], health behaviour), post-migration factors (e.g. host countries contextual factors, health systems and policies and lifestyle changes), genetics and shared family habits can influence socioeconomic circumstances, behaviour and biological factors, access to healthcare, physical and psychosocial stress and epigenetics upon migration, which in turn affect insulin secretion and action and subsequently type 2 diabetes risk [33]. Because of the limited data on genetics and gene–environment interactions in relation to type 2 diabetes among migrants in Europe, we discuss only pre-migration and post-migration factors in detail.

### Post-migration factors

Migration is associated with huge changes in SES, lifestyle and wellbeing of migrants. One of the important changes upon migration to a new country is adjustment to the new environment. This involves changes in dietary habits, physical activity levels, psychosocial stress and socioeconomic circumstances, which are important risk factors for type 2 diabetes [33]. While migration can be beneficial in terms of improvement in SES, it can also be disastrous in terms of unhealthy lifestyle, increased psychosocial stress and limited access to preventive and curative care, with a major impact on biological factors such as obesity, IFG and subsequent type 2 diabetes [34].

Western diet, characterised by high consumption of red meat, processed meat, sweets, desserts and high-fat dairy products, has been shown to be associated with an increased risk of type 2 diabetes [35]. Evidence indicates changes in dietary behaviour upon migration [36, 37], which may contribute to the high prevalence of type 2 diabetes among migrants. Garduño-Diaz and Khokhar’s study on the association between dietary patterns and the metabolic syndrome among South Asians living in the UK found a direct association between the western-pattern diet and the overall risk of the metabolic syndrome [36]. Furthermore, a sedentary lifestyle reduces energy expenditure, promotes weight gain and increases type 2 diabetes risk [38]. Changes in lifestyle (e.g. changes in body weight) upon migration can influence health, including type 2 diabetes. The RODAM study, which assessed African migrants living in three European countries (England, the Netherlands and Germany) and their non-migrant compatriots living in Africa, showed that the prevalence of obesity among African (Ghanaian) migrant men in Europe was up to 15 times higher than in their compatriots living in rural Ghana [17]. The high prevalence of obesity in Ghanaian migrants was consistent with the higher prevalence of type 2 diabetes in migrants compared with their rural Ghanaian peers [17].

Migrants in European cities are frequently congregated in deprived neighbourhoods, where opportunities for social integration, as well as access to healthy food options, a safe walking environment, social support systems, healthcare providers and quality of care are often limited. This disadvantageous position makes it difficult for many migrants to engage in healthy lifestyles and can subsequently impact their health risk. White et al’s natural experiment among refugees in Sweden showed that refugees who were dispersed to highly deprived neighbourhoods had a 22% higher risk of diabetes compared with refugees who were dispersed to low-deprivation neighbourhoods [39].

Psychosocial stress and depression are associated with an increased risk of type 2 diabetes [40]. Many migrants experience discrimination, risk of deportation, separation from families, poor social circumstances including ‘3D’ (dirty, dangerous and demeaning) jobs, poor housing, poor quality of sleep and pressure from their home countries to remit money to support those left behind; such difficulties may expose them to a high risk of psychosocial stress and depression. This, in turn, increases their risk of type 2 diabetes [41, 42]. In the HELIUS study in the Netherlands, the prevalence of self-reported perceived discrimination ranged from 28% in Ghanaian migrants to 31% in Turkish migrants, compared with 2% in European Dutch [41, 42]. The high prevalence of perceived discrimination among these migrants groups was associated with obesity [43] and the metabolic syndrome [44], important risk factors for type 2 diabetes.

There is marked variation between host countries in terms of food environment, politics of migration, ethnic relations and organisation of healthcare and preventive services, and this can have a profound impact on the health of migrants both directly and indirectly. These differential contexts can influence migrants’ health behaviour, socioeconomic circumstances, psychosocial stress and access to preventive services and healthcare and subsequent risk of diseases including type 2 diabetes [45]. Studies comparing similar South Asian Indian and African-Caribbean residents in England and the Netherlands found very important differences in obesity and type 2 diabetes among these groups living in these two countries [45, 46]. Furthermore, the risk for type 2 diabetes is higher in women who have had gestational diabetes than in those who have not, due to defects in both insulin secretion and insulin action [47]. Evidence suggests that the prevalence of gestational diabetes is higher in most migrant groups than in Europeans, which may contribute to their high risk of type 2 diabetes [48].

### Pre-migration factors

Pre-migration factors may also contribute to the high prevalence of type 2 diabetes among migrants. Evidence suggests that adverse early life factors such as low birthweight and malnutrition have a profound effect on several health outcomes including type 2 diabetes in adulthood. Low childhood SES and undernutrition during early development have been shown to be associated with an increased risk of type 2 diabetes [49]. This is thought to be related to the theory of ‘thrifty genotype’ in which insulin resistance might improve survival rate during states of energy scarcity but would lead to type 2 diabetes in states of energy excess and the theory of ‘thrifty phenotype’ in which malnutrition during foetal and early life may be associated with an increased risk of insulin resistance, glucose intolerance and type 2 diabetes in adulthood [50]. Many migrants from poor-resource countries have to some extent been exposed to poor nutritional circumstances during early life, such as low birthweight, malnutrition and stunting due to poor nutrition driven by poor socioeconomic circumstances, which may expose them to high risk of type 2 diabetes upon migration to a food-abundant environment in Europe [51]. Furthermore, pre-migration psychosocial stress (often resulting from poverty, wars and human rights violation, which force many to flee their home countries) can have a negative impact on health, including type 2 diabetes, after migration to Europe. The migration process itself can also be treacherous, with a devastating impact on psychological wellbeing among migrants, especially among asylum seekers. According to the United Nations High Commissioner for Refugees (UNHCR), migrants routinely face horrors during their journeys such as starvation, dehydration, lack of access to medical care, arbitrary detention, kidnapping, trafficking and sexual abuse [52, 53]. The horrible experiences of some migrants en route can leave psychological scars and subsequently have an impact on their physical health, including type 2 diabetes, directly or indirectly through adoption of risky behaviour such as smoking, alcoholism or sedentary lifestyles as coping strategies. A study among asylum seekers in the Netherlands found that individuals with post-traumatic stress disorder (PTSD) had higher odds of diabetes than individuals without PTSD [54].

## Prevention and treatment of type 2 diabetes in migrants

### Prevention

Though the lifetime risk of developing type 2 diabetes is high, it is difficult to predict and prevent type 2 diabetes in the general population. Nevertheless, individuals at high risk of type 2 diabetes, such as those who are obese, those with IFG or impaired glucose tolerance (IGT) and some migrant groups originating from South Asia, the Middle East and Africa, are appropriate candidates for preventive interventions [55]. Lifestyle intervention involving weight loss and increasing physical activity levels can improve insulin sensitivity and glucose tolerance and can prevent progression from IGT to type 2 diabetes [56]. However, evidence indicates that it is difficult to achieve sustained weight management with or without increased physical activity [57]. Furthermore, it has been well demonstrated that community engagement interventions have a positive effect on health behaviours, health consequences, self-efficacy and perceived social support outcomes across various health conditions [58]. However, most intervention trials have been conducted among non-migrant populations, and it remains uncertain whether the results from these trials can be extrapolated to migrants with different lifestyle and cultural traditions. Thus, evidence on how best to deliver effective culturally adapted health promotion interventions to prevent type 2 diabetes, such as physical activity and healthy eating, to migrant groups in Europe is limited [59, 60]. In the last few years, however, culturally adapted lifestyle modification intervention trials to prevent type 2 diabetes in high-risk South Asian adults have been conducted in Norway [61], Scotland [62], the Netherlands [63] and in Iraqi migrants in Sweden [64]. The results suggest more modest effects [65] compared with trials in Europeans [66]. In addition, evidence on the clinical effectiveness or cost-effectiveness, as well as the long-term effect, of these adapted health promotion interventions among migrants in Europe is currently lacking [67]. These observations clearly demonstrate the need to identify the key facilitators and barriers for migration-related lifestyle changes, taking into account national context [68], to support cultural adaptation and evaluation of the effectiveness of these interventions among migrant groups. Moreover, other major migrant groups, such as those of African and East Asian descent, that have an increased risk of type 2 diabetes should be included in these intervention trials.

### Treatment

Evidence suggests that culturally appropriate education intervention can lead to improvement in glycaemic control among migrants with diabetes. A meta-analysis of 28 randomised controlled trials, including four studies in South Asians from the UK and one study from the Netherlands, on culturally appropriate diabetes health education interventions showed significant improvements in glycaemic control and diabetes knowledge over a period of 24 months among the intervention group compared with those receiving usual care [60]. However, evidence suggests that there is poor glycaemic control among migrant groups in Europe despite their high levels of diabetes awareness and treatment [69–71]. In one Dutch study, the awareness and medical treatment of type 2 diabetes were two to five times higher in migrants than in European Dutch individuals, although levels of awareness/treatment in those with glycaemic control (HbA_1c_ levels on target ≤52 mmol/mol) ranged from 37 to 53% in migrant men compared with 67% in European Dutch men [9]. A study in Scotland also found higher rate of suboptimal glycaemic control in migrants compared with European Scots despite migrants being generally younger and having lower BMI [72]. The poor glycaemic control among migrants has been suggested to be due to poor adherence to treatment and lifestyle recommendations, possibly due to low health literacy, poor care standards, poor quality of care or ineffective response to glucose-lowering agents [9]. Furthermore, a large proportion of individuals who migrate from the Middle East and South Asia to Europe have insulin-deficient diabetes, which may also contribute to their poor diabetes control [13, 25]. These observations suggest the need for greater efforts to improve the effectiveness of type 2 diabetes treatment in migrant groups in Europe, taking into account the migrants’ personal perspectives on diabetes. This is highly relevant because a systematic review of various ethnic minority patients’ views on self-management of type 2 diabetes identified several analytical themes that highlight the complex nature of self-management of type 2 diabetes among these populations [73]. How patients identified themselves and the feeling of being understood by health professionals about their culture and by family members regarding their condition were of great importance to successful self-management, while patients’ own limited understanding of diabetes was a barrier to self-management of their condition [73].

## Conclusions and recommendations

Type 2 diabetes and related microvascular and macrovascular complications remain a major burden among migrant populations in Europe. However, the extent of the burden varies across migrant groups and the countries in which they now live. Earlier studies found higher mortality rates among migrants, but recent studies seem to suggest a shifting trends in favour of migrants. Explanations for the high risk of type 2 diabetes among migrants in Europe are mainly speculative. Evaluation of diabetes interventions among migrants are limited and mainly focus on South Asian populations. The level of awareness of diabetes among migrant populations is high, but glycaemic control remains suboptimal relative to Europeans. These observations call for investment in prospective studies and basic scientific research to gain insight into the causal pathways linking migration to the development of type 2 diabetes. The research should include the role of genetics, epigenetics, early life factors, key specific migration-related lifestyle changes and psychosocial stressors. There is also a need for intervention trials of longer duration to investigate clinical outcome measures such as development of microvascular and macrovascular complications and to evaluate the cost-effectiveness of the adapted interventions. The current data on the culturally adapted lifestyle modification intervention trials to prevent type 2 diabetes are mainly based on South Asian adults in Europe, suggesting the need for more work among other major migrant groups such as African-Caribbeans, Sub-Saharan Africans, North Africans, East Asian and Middle Eastern populations, such as Turkish and Iraqi populations, in whom type 2 diabetes is also highly prevalent. Lastly, in order to improve type 2 diabetes treatment outcomes among migrant groups in Europe, more work is needed to gain better understanding of factors driving the poor treatment outcomes and to help develop cultural tailored interventions for migrants with type 2 diabetes.

## Supplementary information

Slideset of figures (PPTX 400 kb)

## Abbreviations

CHD: Coronary heart disease
HELIUS: Healthy Life in an Urban Setting
IFG: Impaired fasting glucose
PTSD: Post-traumatic stress disorder
RODAM: Research on Obesity and Diabetes among African Migrants
SES: Socioeconomic status
