# Supplementary material for: Type 2 diabetes burden among migrants in Europe: unravelling the causal pathways
Source: Diabetologia. 2021 Oct 16;64(12):2665–75. doi: 10.1007/s00125-021-05586-1 (PMC8563673; doi:10.1007/s00125-021-05586-1)
Supplement: Supplementary file 1 — (PPTX 400 kb) [file 125_2021_5586_MOESM1_ESM.pptx]

## Slide 1
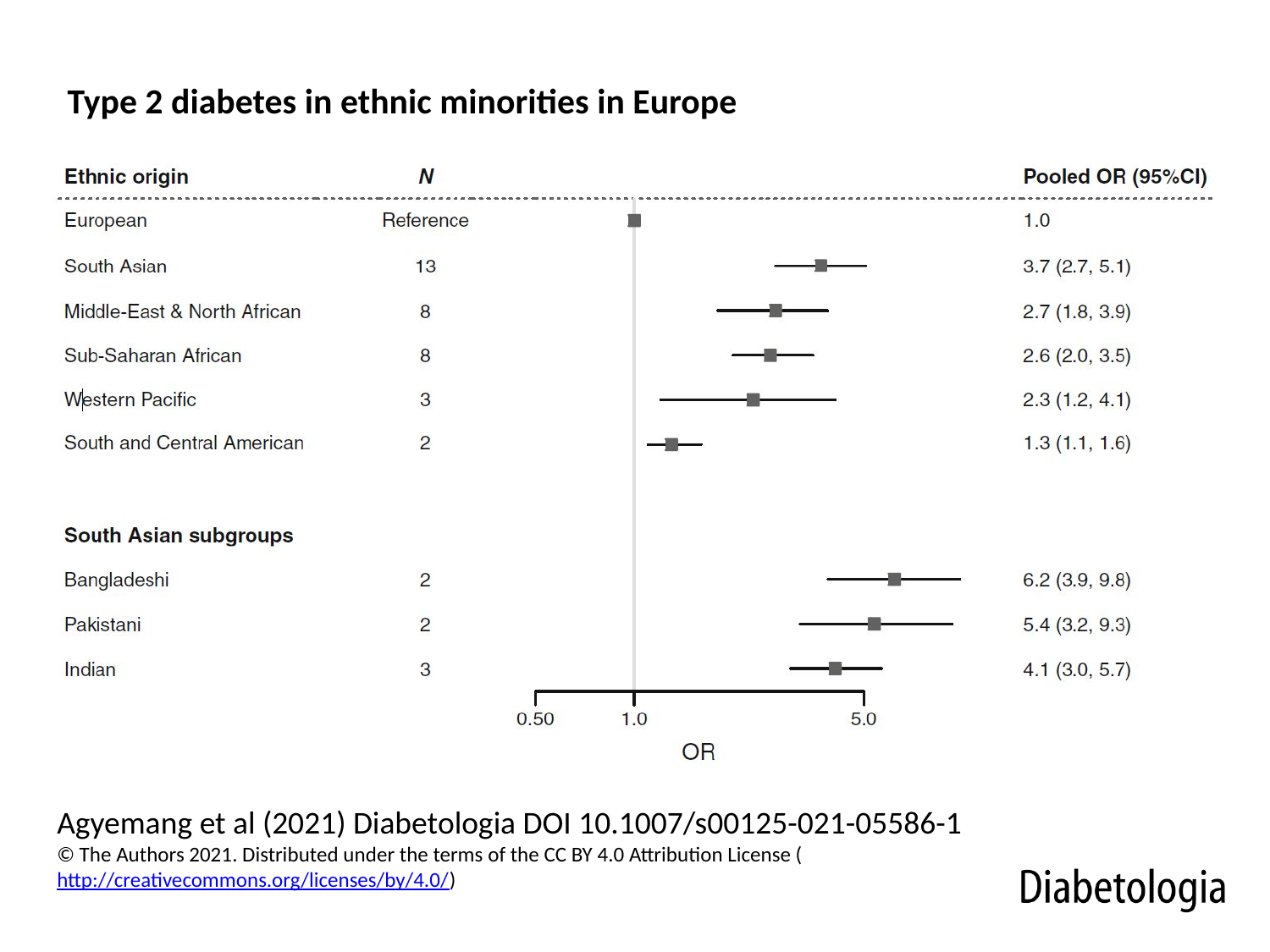

Type 2 diabetes in ethnic minorities in Europe
Agyemang et al (2021) Diabetologia DOI 10.1007/s00125-021-05586-1
© The Authors 2021. Distributed under the terms of the CC BY 4.0 Attribution License (http://creativecommons.org/licenses/by/4.0/)

## Slide 2
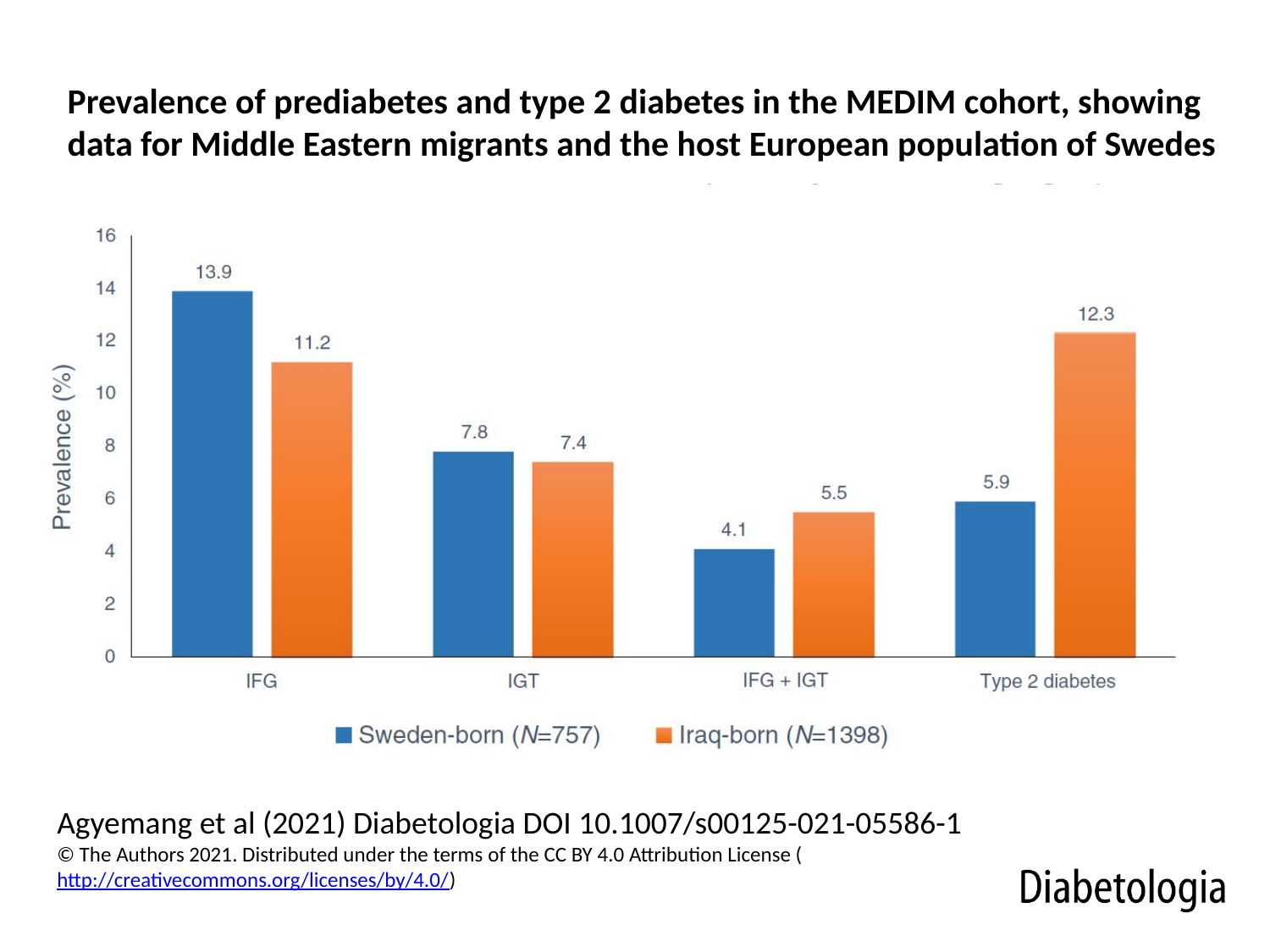

Prevalence of prediabetes and type 2 diabetes in the MEDIM cohort, showing data for Middle Eastern migrants and the host European population of Swedes
Agyemang et al (2021) Diabetologia DOI 10.1007/s00125-021-05586-1
© The Authors 2021. Distributed under the terms of the CC BY 4.0 Attribution License (http://creativecommons.org/licenses/by/4.0/)

## Slide 3
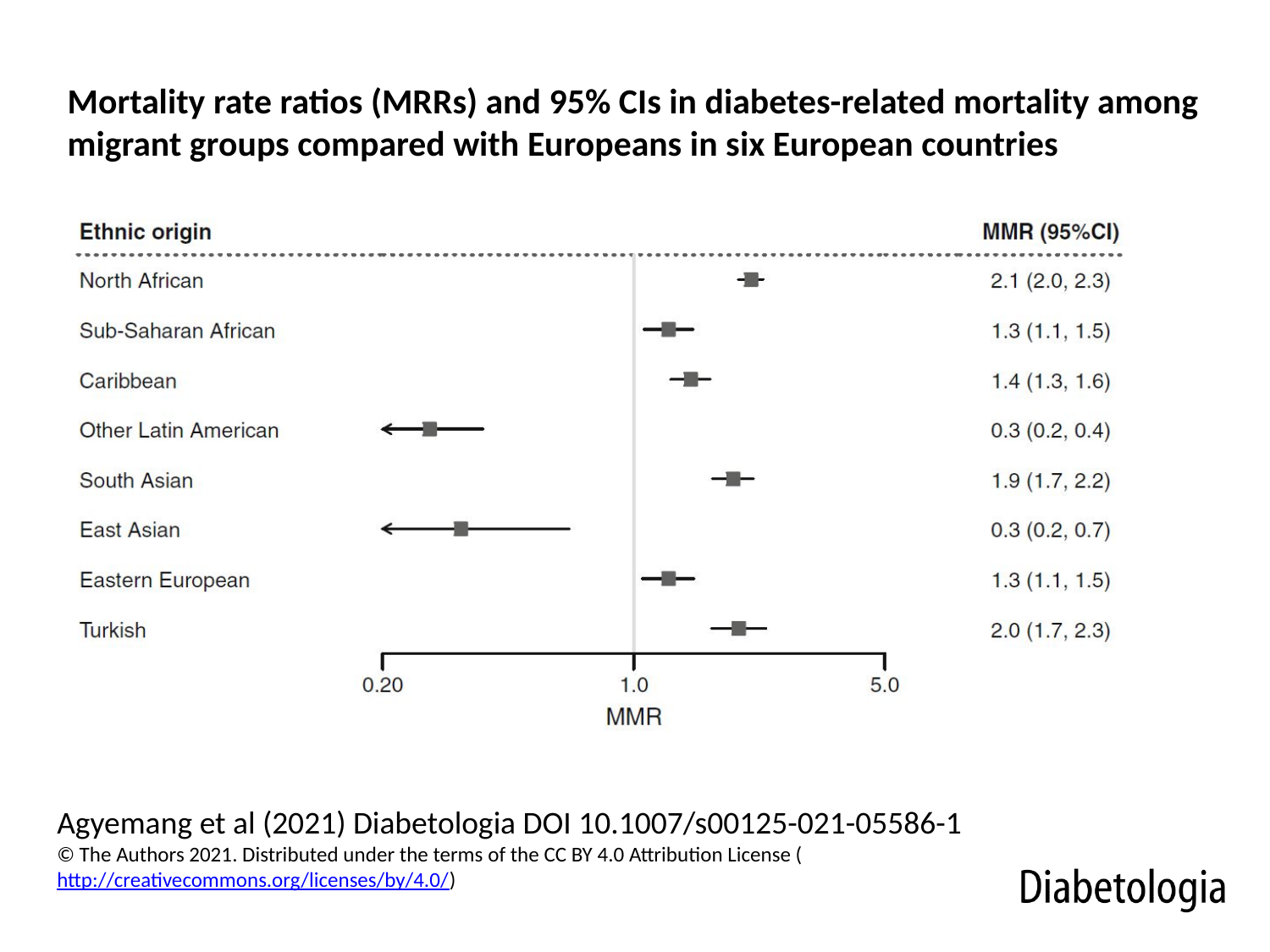

Mortality rate ratios (MRRs) and 95% CIs in diabetes-related mortality among migrant groups compared with Europeans in six European countries
Agyemang et al (2021) Diabetologia DOI 10.1007/s00125-021-05586-1
© The Authors 2021. Distributed under the terms of the CC BY 4.0 Attribution License (http://creativecommons.org/licenses/by/4.0/)

## Slide 4
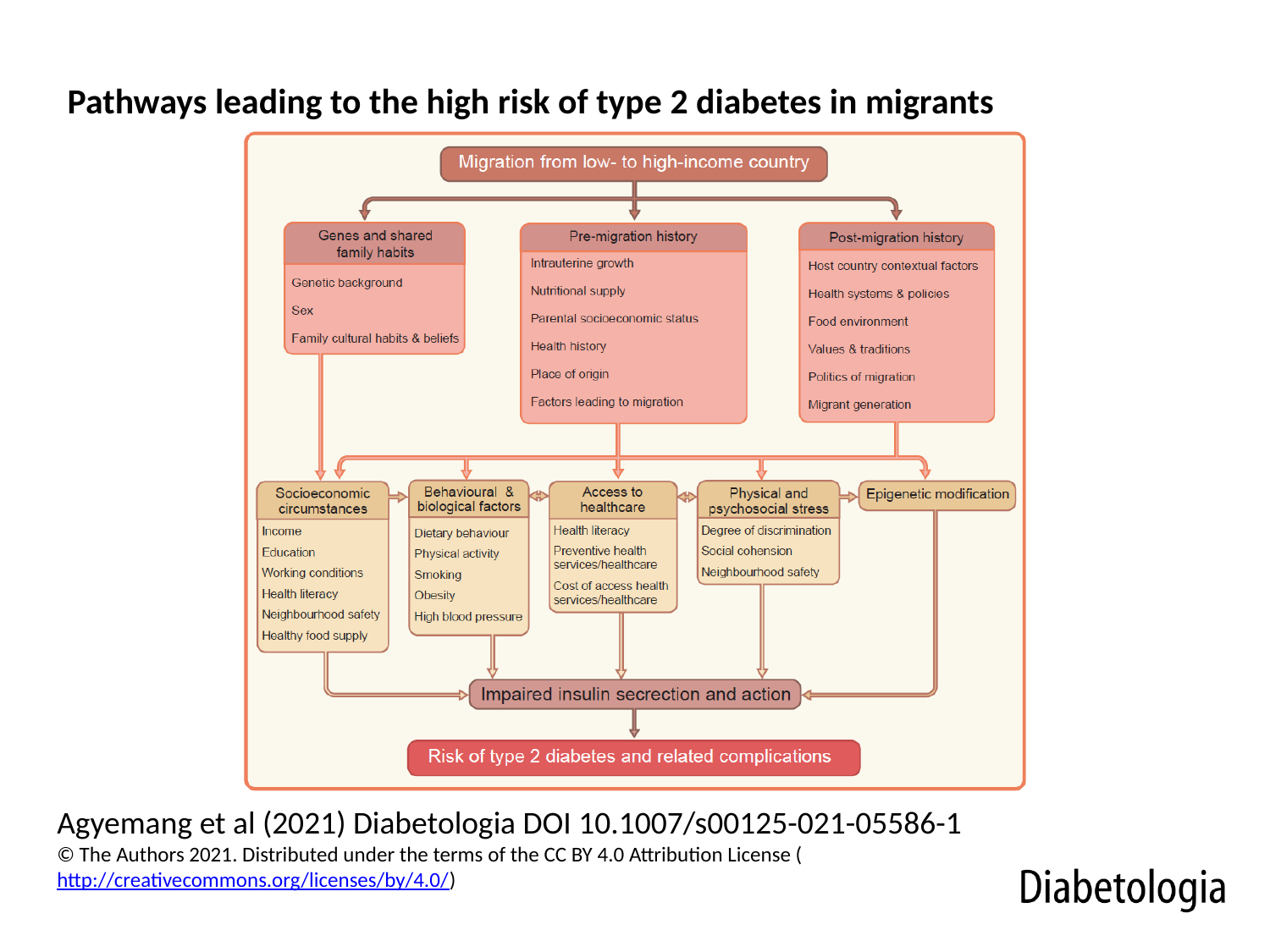

Pathways leading to the high risk of type 2 diabetes in migrants
Agyemang et al (2021) Diabetologia DOI 10.1007/s00125-021-05586-1
© The Authors 2021. Distributed under the terms of the CC BY 4.0 Attribution License (http://creativecommons.org/licenses/by/4.0/)
